# Fuzzy Logic-Based Adaptive Filtering for Transfer Alignment

**DOI:** 10.3390/s25164998

**Published:** 2025-08-12

**Authors:** Zhaohui Gao, Jiahui Yang, Chengfan Gu, Yongmin Zhong

**Affiliations:** 1 School of Electronic Engineering, Xi’an Shiyou University, Xi’an 710065, China; gzhgzhstation@hotmail.com; 2 School of Automatics, Northwestern Polytechnical University, Xi’an 710072, China; yjhyjh@mail.nwpu.edu.cn; 3 School of Engineering, RMIT University, Bundoora, VIC 3083, Australia; chengfan.gu@iinet.net.au

**Keywords:** transfer alignment, adaptive robust filtering, strapdown inertial navigation, fuzzy logic theory

## Abstract

The transfer alignment of strapdown inertial navigation systems (SINSs) is of great significance for improving the strike accuracy of airborne tactical vehicles. This study designed a new fuzzy logic-based adaptive filtering method by using the fuzzy logic theory to address the influence of system model error on the state estimation of the Kalman filter for SINS transfer alignment. It established the state error model and measurement error model, which were embedded with the state prediction residual and measurement residual, respectively, for SINS transfer alignment. The fuzzy rules were designed and introduced into the Kalman filtering framework to estimate the covariances of the system measurement and predicted state by minimizing their residuals to improve filtering accuracy for SINS transfer alignment. Simulation and experimentation together with associated comparative analyses were conducted, demonstrating that the proposed method can effectively handle the influence of system model error on SINS transfer alignment, and its accuracy is at least 18.83% higher than benchmark methods for transfer alignment.

## 1. Introduction

Strapdown inertial navigation systems (SINSs) are a mainstream means of navigation and positioning of airborne tactical vehicle systems due to its simple structure, small volume, and convenient maintenance [1,2]. An airborne tactical vehicle is launched by a carrier. Before the launch, the airborne vehicle’s SINS (slave SINS) must be aligned with the carrier’s SINS (master SINS), leading to transfer alignment. Transfer alignment refers to comparing the navigation information of an airborne vehicle’s slave SINS with that of the high-precision master SINS on the carrier to estimate the relative misalignment angles between the slave and master SINSs [3,4]. If the transfer alignment is inaccurate, the airborne vehicle will fail to strike the target and may even deviate far from it. The key to transfer alignment is to establish an appropriate transfer alignment mathematical model and associated filtering algorithm to correct alignment errors [5,6].

Extensive research efforts have been dedicated to transfer alignment, resulting in a variety of transfer alignment methods [7,8]. The joint matching of velocity and attitude, namely, fast transfer alignment, uses the Kalman filter to process the velocity and attitude information of the master SINS to estimate the required alignment information [9]. This method can achieve sub-millimeter accuracy for angular alignment in a very short time, even for wing–pendulum maneuvers. Without requiring long-term lateral acceleration, it can achieve fast SINS alignment even in the ocean swaying environment [10,11]. Therefore, the velocity and attitude matching method is widely used for SINS transfer alignment [12,13].

However, the use of the Kalman filter in the velocity and attitude matching method requires an accurate dynamic model [14]. If the system model involves error, the filtering solution will be biased or even divergent [15,16]. However, in engineering practice, the system model is only a theoretical approximation of the actual system and, thus, it inevitably involves error [17,18]. The system model error is different from the sensor measurement error and lever arm error. The measurement error is an intrinsic property of a sensor, which characterizes sensor accuracy. The lever-arm error is related to the distance between the master and slave inertial navigation systems and aircraft maneuverability. These two types of error can be calibrated in advance, while the system model error cannot be calibrated, given that it is an intrinsic attribute of a dynamic system involving uncertainties. Therefore, it is necessary to establish a high-performance filtering algorithm to suppress the influence of system model error to improve transfer alignment accuracy.

Fuzzy logic is a powerful tool to solve uncertain and imprecise problems. It can efficiently handle the influence of random errors and uncertainties on the performance of a dynamic system in the manner of the human brain’s reasoning and thinking, leading to the incomparable capability in solving uncertain and imprecise problems compared to conventional methods [19]. Given the uncertainties of system model error in transfer alignment, fuzzy logic provides a promising solution to address its negative influence on alignment results.

This paper presents a new, fuzzy logic, adaptive filtering method to suppress the influence of system model error on Kalman filtering estimation for transfer alignment. It establishes a transfer alignment error model in terms of position, velocity, and attitude by introducing position information into the velocity–attitude matching method. Based on this, a fuzzy logic adaptive filtering algorithm is developed by combining the fuzzy theory into the Kalman filtering framework to handle the influence of system model error on transfer alignment, where the covariances of system measurement and predicted state are estimated by minimizing their residuals to correct transfer alignment error. Simulation and experimental results together with comparison analyses indicate that the proposed technology can effectively suppress the influence of system model error on transfer alignment, leading to improved performance compared to the Kalman filter and other existing techniques.

## 2. Related Work

Considerable research efforts have been dedicated to studying various errors that affect the accuracy of transfer alignment. These errors include the errors of inertial components, installation error, lever-arm effect, and wing bending deformation effect [20,21,22]. El-Sheimy et al. reported a method to enhance the signal-to-noise ratio of measured signals by preprocessing measurement data [23]. Reiner studied a transfer alignment method to cope with the influence of errors by utilizing less affected measurements [24]. These two methods improve alignment accuracy with the trade-off of alignment time. Sun et al. developed a wavelet cascade denoising technique to reduce the IMU (inertial measurement unit) measurement noise, leading to improved transfer alignment performance [25]. However, this algorithm is based on the spectrum characteristics of IMU raw measurements, leading to the complexity of implementation. Wang and Deng studied a Kalman filter for SINS transfer alignment [26]. This Kalman filter can achieve high filtering accuracy against system noise uncertainty. Cui et al. proposed a second-order extended Kalman filter (EKF) and a unified model for transfer alignment at random misalignment angles [27]. However, these two Kalman filters do not consider the disturbance of system model error and state parameter estimation. Chu et al. employed an adaptive Kalman filter to estimate misalignment angles by considering the flexure effect on transfer alignment [28]. This adaptive Kalman filter reduces the dependence on prior knowledge of system noise statistics, leading to improved filtering performance compared to the standard Kalman filter. However, the adaptive Kalman filter is poor in stability, and its adaptive computational process is also difficult to implement due to the presence of random errors [29,30,31]. Gao et al. reported a robust adaptive Kalman filter (RAKF) for a SINS/SAR (Strap-down Inertial Navigation System/Synthetic Aperture Radar)-integrated navigation system [32,33]. However, with this RAKF, it is difficult to determine the optimal robust and adaptive factors to attain optimal filtering results. Ali and Ushaq introduced a robust Kalman filter for inertial navigation system (INS) transfer alignment [34]. However, this work focuses on using Huber’s generalized maximum likelihood estimation theory to improve the Kalman filter robustness, without considering the effect of system model error on filtering estimation. Gong et al. studied a robust adaptive Student–Kalman filter to address the influence of non-Gaussian system noise on Kalman filtering estimation [35]. Huang et al. reported a slide window variational adaptive Kalman filter to address the influence of inaccurate state and measurement noise covariances on Kalman filtering solutions [36]. Bai et al. investigated a statistical similarity measure-based adaptive Kalman filter to estimate noise covariance in the presence of measurement outliers [37]. Similar to the work by Ali and Ushaq, these adaptive and/or robust filters fail to consider the effect of system model error on state estimation. In general, most existing studies do not consider the interference of system model errors in state estimation.

Fuzzy logic is a universal approximation method that is broadly used to deal with partially or entirely uncertain problems [38]. It enables effective modeling of the uncertainty and imprecision often encountered in an actual dynamic system [39]. Loebis et al. used fuzzy logic to accommodate the initial statistical assumption of both Kalman filter and EKF caused by random variations in sensor noise characteristics [40]. Kobayashi et al. introduced a fuzzy logic-based Kalman filter to improve the positioning accuracy of a differential global positioning system [41]. Sasiadek et al. reported a fuzzy Kalman filter to fuse the position signals from the Global Positioning System (GPS) and INS for the autonomous navigation of mobile vehicles [42]. Hu et al. employed an indirect fuzzy robust cubature–Kalman filter algorithm to address the degraded performance of the cubature Kalman filter (CKF) due to measurement uncertainty [43]. However, the above studies focus on using fuzzy logic to improve vehicle navigation and positioning, while there has been limited research on the use of fuzzy logic to improve transfer alignment accuracy.

Different from the existing studies on transfer alignment, this paper presents a new method for the transfer alignment of an airborne vehicle’s SINS. Its contributions include the following: (i) a new fuzzy logic-based adaptive filtering algorithm is developed by combining the fuzzy logic theory with Kalman filtering for SINS transfer alignment; (ii) the error models for SINS transfer alignment are established, where the state prediction residual and measurement residual are embedded in the state error model and measurement error model, respectively, to compensate for the influence of system model error; and (iii) fuzzy logic rules are designed and further introduced into the Kalman filtering framework to estimate the covariances of the system measurement vector and predicted state vector by minimizing the residuals.

## 3. Transfer Alignment Error Model

The alignment of an airborne vehicle’s SINSs (slave SINSs) carried by a moving carrier needs position, velocity, and attitude information from the carrier’s SINS (master SINS). The state vector for transfer alignment is represented as

(1) $$X\left(t\right)={\left[\begin{array}{ccccccccccccccc} \delta v_{E} & \delta v_{N} & \delta v_{U} & \phi_{E} & \phi_{N} & \phi_{U} & \delta \varphi & \delta \lambda & \delta \psi & \nabla_{E} & \nabla_{N} & \nabla_{U} & \varepsilon_{E} & \varepsilon_{N} & \varepsilon_{U} \end{array}\right]}^{T}$$

where $\left(\begin{array}{ccc} \delta v_{E} & \delta v_{N} & \delta v_{U} \end{array}\right)$ is the velocity difference between the master and slave SINSs in the East, North, and Up directions; $\left(\phi_{E},\phi_{N},\phi_{U}\right)$ is the attitude angle error of the slave SINS; (δφ, δλ, δψ) is the attitude difference between the master and slave INSs; ($\nabla_{E},\nabla_{N},\;\nabla_{U}$) is the zero bias of the accelerometer in the East, North, and Up directions; and $\left(\varepsilon_{E},\varepsilon_{N},\varepsilon_{U}\right)$ represents gyroscopic drift.

The error model for SINS transfer alignment is described as [44,45]

(2) $$\left\{\begin{array}{l} \frac{d\left(\delta v^{n}\right)}{dt}=f^{n}\times \phi^{n}-(2\omega_{ie}^{n}+\omega_{en}^{n})\times \delta v-(2\delta \omega_{ie}^{n}+\delta \omega_{en}^{n})\times v^{n}+\nabla^{n}\\ \frac{d\phi^{n}}{dt}=-\omega_{in}^{n}\times \phi^{n}+\delta \omega_{ie}^{n}+\delta \omega_{en}^{n}+\varepsilon^{n}\\ \frac{d\left(\delta \varphi \right)}{dt}=\delta v_{N}\frac{1}{R_{M}+h}-\delta h\frac{v_{N}}{{\left(R_{M}+h\right)}^{2}}\\ \frac{d\delta \lambda }{dt}=\delta v_{E}\frac{\sec\varphi }{R_{N}+h}+\delta \varphi \frac{v_{E}\tan\varphi \sec\varphi }{R_{N}+h}-\delta h\frac{v_{E}\sec\varphi }{{\left(R_{M}+h\right)}^{2}}\\ \frac{d\delta h}{dt}=\delta v_{U}\\ \frac{d\varepsilon }{dt}=0,\;\;\;\frac{d\nabla }{dt}=0 \end{array}\right.$$

where (n,i,e) denotes the navigation frame (*n*-frame), inertial frame (*i*-frame), and Earth frame (*e*-frame) and the *n*-frame systems of both master and slave SINSs are the E-N-U (Earth–North–Up) geographic frame system. $\omega_{ie}^{n}$ and $\omega_{en}^{n}$ denote the angular rates of the *e*-frame with respect to the *i*-frame and the *n*-frame with respect to the *e*-frame, respectively, and they are expressed in the *n*-frame. $f^{n}$ is the specific force of SINS expressed in the *n*-frame. $\phi^{n}$ is the misalignment angle between the master and slave SINSs expressed in the *n*-frame. ε and ∇ are the gyro and accelerometer biases. Both $R_{M}$ and $R_{N}$ are the curvature radiuses. v is the velocity vector with $v_{E},\;v_{N}$, and $v_{U}$ as components in the East, North, and Up directions. φ, λ, and *h* represent the latitude, longitude, and altitude of the aircraft.

According to (1) and (2) and considering that the system involves noise, the system state equation for transfer alignment can be established as

(3) $$\dot{X}\left(t\right)=F\left(X\left(t\right),t\right)+w\left(t\right)$$

where $X\left(t\right)$ is the system state, $F\left(X\left(t\right),t\right)$ is the nonlinear state function, and $w\left(t\right)$ is the process noise.

By linearizing the nonlinear state function $F\left(X\left(t\right),\;t\right)$ using Taylor expansion, (3) can be further written as

(4) $$\dot{X}\left(t\right)=\Phi \left(t\right)X\left(t\right)\;+w\left(t\right)$$

where $\Phi \left(t\right)={\left.\frac{\partial F\left(X(t),t\right)}{\partial X(t)}\right|}_{X(t)=\hat{X}(t_{k-1})}$ and $\hat{X}(t_{k-1})$ is the estimated state at time $t_{k-1}$.

The system measurement vector is defined as

(5) $$Z(t)={\left[\begin{array}{cccccc} \delta v_{E} & \delta v_{N} & \delta v_{U} & \delta \varphi & \delta \lambda & \delta \psi \end{array}\right]}^{T}$$

Considering that the system measurement involves noise, the measurement equation for transfer alignment can be described as

(6) $$Z\left(t\right)=A\left(t\right)X\left(t\right)+e\left(t\right)$$

where $e\left(t\right)$ is the measurement noise and $A\left(t\right)$ is the measurement matrix, which is given as

(7) $$A\left(t\right)=\left[\begin{array}{ccc} I_{2\times 2} & 0_{2\times 3} & 0_{2\times 10}\\ 0_{2\times 2} & H_{2\times 3} & 0_{2\times 10} \end{array}\right]$$

with **H** as the MSINS attitude matrix.

The system models described by (4) and (5) are only an approximation of the actual system, leading to system model errors. Considering system model errors, (4) and (5) can be further written as

(8) $$\left\{\begin{array}{l} \dot{X}\left(t\right)=\Phi \left(t\right)X\left(t\right)+B\left(t\right)s\left(t\right)+w\left(t\right)\\ Z\left(t\right)=A\left(t\right)X\left(t\right)+G\left(t\right)u\left(t\right)+e\left(t\right) \end{array}\right.$$

where $s\left(t\right)$ and $u\left(t\right)$ are the process model error and measurement model error and $B\left(t\right)$ and $G\left(t\right)$ are their coefficient matrices.

Discretizing (8) in the time domain yields

(9) $$\left\{\begin{array}{l} X_{k}=\Phi_{k,k-1}X_{k-1}+B_{k,k-1}s_{k}+w_{k}\\ Z_{k}=A_{k}X_{k}+G_{k}u_{k}+e_{k} \end{array}\right.$$

For simplification and without the loss of generality, it is assumed that $B_{k,k-1}=I$, $G_{k}=I$, $w_{k}$, and $e_{k}$ are the Gauss noises with expectations $E(w_{k})=0$ and $E(e_{k})=0$ as well as covariances $Q_{k}=\Sigma_{w_{k}}=E\left(w_{k}w_{k}^{T}\right)$ and $R_{k}=\Sigma_{e_{k}}=E\left(e_{k}e_{k}^{T}\right)$; $s_{k}$, $u_{k}$, $w_{k}$, and $e_{k}$ are independent of each other.

**Figure 1:**
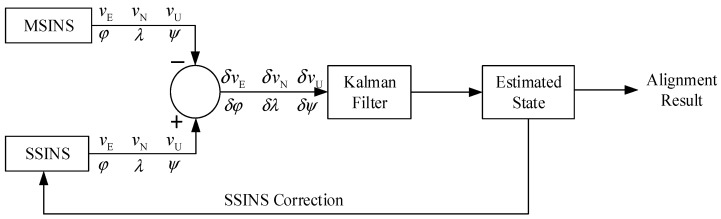
Transfer alignment framework.

Equation (9) provides the system state and measurement equations by taking into account the system model errors. Based on this, this study developed a new Kalman filter to suppress the interference of the system model errors on the Kalman filtering solution for transfer alignment. Figure 1 shows the transfer alignment framework, the core of which is the Kalman filter. The velocity and attitude errors between MSINS and SSINS outputs are input as the measurements into the Kalman filter. By Kalman filtering estimation, the Kalman filter outputs the estimated state, which is further used to correct the SSINS output. After recursive filtering iterations, the estimated state generated by the Kalman filter is eventually taken as the alignment result to complete the transfer alignment between MSINS and SSINS.

## 4. Fuzzy Logic-Based Adaptive Filtering Algorithm

The predicted state ${\bar{X}}_{k}$ is expressed as

(10) $${\bar{X}}_{k}=\Phi_{k,k-1}{\hat{X}}_{k-1}$$

where ${\hat{X}}_{k-1}$ is the estimated state value at time $t_{k-1}$.

The predicted state’s residual is defined as

(11) $$V_{{\bar{X}}_{k}}={\hat{X}}_{k}-{\bar{X}}_{k}={\hat{X}}_{k}-\Phi_{k,k-1}{\hat{X}}_{k-1}$$

where ${\hat{X}}_{k}$ is the estimated state and ${\bar{X}}_{k}$ is the one-step predicted state.

Applying the covariance propagation law to (11) yields

(12) $$\begin{array}{l} \Sigma_{V_{{\bar{X}}_{k}}}=E[({\hat{X}}_{k}-{\bar{X}}_{k})-E({\hat{X}}_{k}-{\bar{X}}_{k})]{\left[({\hat{X}}_{k}-{\bar{X}}_{k})-E({\hat{X}}_{k}-{\bar{X}}_{k})\right]}^{T}\\ \;\;\;\;\;\;\;\;\;=E\{[{\hat{X}}_{k}-E({\hat{X}}_{k})]-[{\bar{X}}_{k}-E({\bar{X}}_{k})]\}{\left\{[{\hat{X}}_{k}-E({\hat{X}}_{k})]-[{\bar{X}}_{k}-E({\bar{X}}_{k})]\right\}}^{T}\\ \;\;\;\;\;\;\;\;\;=\Sigma_{{\hat{X}}_{k}}+\Sigma_{{\bar{X}}_{k}}-E[{\hat{X}}_{k}-E({\hat{X}}_{k})]{\left[{\bar{X}}_{k}-E({\bar{X}}_{k})\right]}^{T}\\ \;\;\;\;\;\;\;\;\;\;\;-E[{\bar{X}}_{k}-E({\bar{X}}_{k})]{\left[{\hat{X}}_{k}-E({\hat{X}}_{k})\right]}^{T}\\ \;\;\;\;\;\;\;\;\;=\Sigma_{{\bar{X}}_{k}}-\Sigma_{{\hat{X}}_{k}} \end{array}$$

where $\Sigma_{V_{{\bar{X}}_{k}}}$, $\Sigma_{{\bar{X}}_{k}}$, and $\Sigma_{{\hat{X}}_{k}}$ are the covariance matrices of predicted state residual $V_{{\bar{X}}_{k}}$, predicted state ${\bar{X}}_{k}$, and estimated state ${\hat{X}}_{k}$ at time $t_{k}$, respectively.

Considering the estimate of the state model error, the error equation for the predicted state can be written as

(13) $${V^{\prime }}_{{\bar{X}}_{k}}={\hat{X}}_{k}-{\bar{X}}_{k}-{\hat{s}}_{k}$$

where ${V^{\prime }}_{{\bar{X}}_{k}}$ is the predicted residual, which is the residual of predicted state ${\bar{X}}_{k}$ in the presence of state model error, and ${\hat{s}}_{k}$ is the estimate of $s_{k}$.

The measurement vector’s residual is defined as

(14) $$V_{k}=A_{k}{\hat{X}}_{k}-Z_{k}$$

where ${\hat{X}}_{k}$ is the estimated state and $Z_{k}$ is the measurement at time $t_{k}$.

Applying the covariance propagation law to (13), we have

(15) $$\begin{array}{l} \Sigma_{V_{k}}=E[(A_{k}{\hat{X}}_{k}-Z_{k})-E(A_{k}{\hat{X}}_{k}-Z_{k})]{\left[(A_{k}{\hat{X}}_{k}-Z_{k})-E(A_{k}{\hat{X}}_{k}-Z_{k})\right]}^{T}\\ \;\;\;\;\;\;\;\;\;\;\;=E\left(\left\{[A_{k}{\hat{X}}_{k}-A_{k}E({\hat{X}}_{k})]-[Z_{k}-E(Z_{k})]\right\}\right.\\ \;\;\;\;\;\;\;\;\;\;\;\;\;\;\;\times \left.{\left\{[A_{k}{\hat{X}}_{k}-A_{k}E({\hat{X}}_{k})]-[Z_{k}-E(Z_{k})]\right\}}^{T}\right)\\ \;\;\;\;\;\;\;\;\;\;\;=A_{k}\Sigma_{{\hat{X}}_{k}}A_{k}^{T}+\Sigma_{k}-E[A_{k}{\hat{X}}_{k}-A_{k}E({\hat{X}}_{k})]{\left[Z_{k}-E(Z_{k})\right]}^{T}\\ \;\;\;\;\;\;\;\;\;\;\;\;\;\;\;\;-E[Z_{k}-E(Z_{k})]{\left[A_{k}{\hat{X}}_{k}-A_{k}E({\hat{X}}_{k})\right]}^{T}\\ \;\;\;\;\;\;\;\;\;\;\;=A_{k}\Sigma_{{\hat{X}}_{k}}A_{k}^{T}+\Sigma_{k}-E[A_{k}{\hat{X}}_{k}-A_{k}E({\hat{X}}_{k})]{\left[A_{k}X_{k}+e_{k}-A_{k}E(X_{k})\right]}^{T}\\ \;\;\;\;\;\;\;\;\;\;\;\;\;\;\;\;\;\;-E[A_{k}X_{k}+e_{k}-A_{k}E(X_{k})]{\left[A_{k}{\hat{X}}_{k}-A_{k}E({\hat{X}}_{k})\right]}^{T}\\ \;\;\;\;\;\;\;\;\;\;\;=\Sigma_{k}-A_{k}\Sigma_{{\hat{X}}_{k}}A_{k}^{T} \end{array}$$

where $\Sigma_{V_{k}}$, $\Sigma_{k}$, and $\Sigma_{{\hat{X}}_{k}}$ are the covariance matrices of measurement residual $V_{k}$, measurement $Z_{k}$, and estimated state ${\hat{X}}_{k}$ at time $t_{k}$.

Considering the estimate of the measurement model error, the error equation for the measurement residual is written as

(16) $${V^{\prime }}_{k}=A_{k}{\hat{X}}_{k}-Z_{k}-{\hat{u}}_{k}$$

where ${V^{\prime }}_{k}$ is the measurement residual, which is the residual of measurement vector $Z_{k}$ in the presence of the measurement model error, and ${\hat{u}}_{k}$ is the estimate of $u_{k}$.

Assume that there are N measurements within the time period $(t_{k-N}$~$t_{k-1})$. By (13), the residual corresponding to predicted state ${\bar{X}}_{k-i}$ (i=1,⋯,N) is

(17) $${V^{\prime }}_{{\bar{X}}_{k-i}}={\hat{X}}_{k-i}-{\bar{X}}_{k-i}-{\hat{s}}_{k}$$

and its covariance is

(18) $$\begin{array}{l} \Sigma_{{V^{\prime }}_{{\bar{X}}_{k-i}}}=\frac{1}{N}E(\sum_{i=1}^{N}{V^{\prime }}_{{\bar{X}}_{k-i}}{V^{\prime }}_{{\bar{X}}_{k-i}}^{T})\\ \;\;\;\;\;\;\;\;\;\;=\frac{1}{N}E(\sum_{i=1}^{N}\left({\hat{X}}_{k-i}-{\bar{X}}_{k-i}-s_{k}\right)\;{\left({\hat{X}}_{k-i}-{\bar{X}}_{k-i}-s_{k}\right)}^{Τ} \end{array}$$

By (16), the residual of the measurement vector $Z_{k-i}$ can be written as

(19) $${V^{\prime }}_{k-i}=A_{k-i}{\hat{X}}_{k-i}-Z_{k-i}-{\hat{u}}_{k}$$

and its covariance is

(20) $$\begin{array}{l} \Sigma_{{V^{\prime }}_{k}}=\frac{1}{N}E(\sum_{i=1}^{N}{V^{\prime }}_{k-i}{V^{\prime }}_{k-i}^{T})\\ \;\;\;\;\;\;\;\;=\frac{1}{N}E(\sum_{i=1}^{N}\left(A_{k-i}{\hat{X}}_{k-i}-Z_{k-i}-{\hat{u}}_{k}\right){\left(A_{k-i}{\hat{X}}_{k-i}-Z_{k-i}-{\hat{u}}_{k}\right)}^{Τ} \end{array}$$

In fact, the filtering accuracy can be improved by taking full advantage of useful measurement information to resist the disturbance of system model error on system state estimation. Based on this concept, a fuzzy logic algorithm is developed and further combined into the Kalman filtering framework to resist the influence of system model errors $s_{k}$ and $u_{k}$ by minimizing the means and covariances of residuals ${V^{\prime }}_{k}$ and ${V^{\prime }}_{{\bar{X}}_{k}}$. This fuzzy logic-based filtering algorithm contains the following steps:

**Step 1.** Define the input and output of the fuzzy rules as

(21) $$\left\{\begin{array}{l} x_{1}=\frac{1}{N}\sum_{i=1}^{N}V_{k-i}\\ x_{2}=\frac{1}{N}\sum_{i=1}^{N}V_{{\bar{X}}_{k-i}}\\ x_{3}=\Sigma_{V_{k}}\\ x_{4}=\Sigma_{V_{{\bar{X}}_{k}}} \end{array}\right.$$

and

(22) $$\left\{\begin{array}{l} y_{1}={\hat{\Sigma }}_{k}\\ y_{2}={\hat{\Sigma }}_{{\bar{X}}_{k}} \end{array}\right.$$

where $x_{i}(i=1,2,3,4)$ and $y_{j}(j=1,2)$ are the input and output of the fuzzy rules and ${\hat{\Sigma }}_{k}$ and ${\hat{\Sigma }}_{{\bar{X}}_{k}}$ are the covariances of the measurement and predicted state generated by the fuzzy logic rules.

**Step 2**. Fuzzify the input quantity $x_{i}(i=1,2,3,4)$ and the output quantity $y_{j}(j=1,2)$ into fuzzy variables. The input to be determined is transformed into a fuzzy set described by membership degrees. Specifically, the fuzzy subsets of the input variables are classified into three categories (too large, relatively large, and normal) and the fuzzy subsets of the output variables are classified into three categories (too small, relatively small, and normal).

**Step 3**. Imitate human perceptual thinking and reasoning and design fuzzy rules using input $x_{i}(i=1,2,3,4)$ and output $y_{j}(j=1,2)$ as follows:

The means $x_{i}(i=1,2,3,4)$ and covariances $y_{j}(j=1,2)$ given by (21) and (22) can be obtained by observing the residuals of the measurement vector and predicted state.

(1) If the covariance of the measurement residual increases, that is, if the mean deviates from zero, then the state estimation value in (14) will be reduced to minimize the mean deviation.

(2) If the covariance of the predicted state residuals increases, that is, if its mean deviates from zero, then the state estimation value in (11) will be reduced to minimize the mean deviation, thereby minimizing the influence of the measurement residual and predicted state residual on the state estimation.

**Step 4**. Establish the membership functions by calculating the measurement residual mean

(23) $$E\left(V_{k}\right)=\frac{1}{N}\sum_{i=1}^{N}E\left(A_{k}{\hat{X}}_{k}-Z_{k}\right)$$

1. and the predicated state residual’s covariance

(24) $$\Sigma_{{\bar{X}}_{k}}=\Sigma_{{\hat{\bar{X}}}_{k}}\;-\Sigma_{{\hat{X}}_{k}}$$

**Step 5.** Defuzzification

As the expectation of the predicted measurement residual is zero, i.e.,

(25) $$E({V^{\prime }}_{k-i})=0$$

We can obtain from (16)

(26) $$\begin{array}{l} {\hat{u}}_{k}=\frac{1}{N}\sum_{i=1}^{N}\left(A_{k-i}{\hat{X}}_{k-i}-Z_{k-i}\right)\\ \;\;\;\;\;\;=\frac{1}{N}\sum_{i=1}^{N}\left(V_{k-i}\right)\\ \;\;\;\;\;\;=x_{1} \end{array}$$

By (15), we have

(27) $$\Sigma_{k-i}=A_{k-i}\Sigma_{{\hat{X}}_{k-i}}A_{k-i}^{T}+\sum_{i=1}^{N}\Sigma_{V_{k-i}}$$

Thus, the output $y_{1}$ of the fuzzy logic can be written as

(28) $$\begin{array}{l} y_{1}=\frac{1}{N}\sum_{i=1}^{N}\Sigma_{k-i}\\ \;\;\;\;\;=\frac{1}{N}(\sum_{i=1}^{N}A_{k-i}\Sigma_{{\hat{X}}_{k-i}}A_{k-i}^{T}+\sum_{i=1}^{N}\Sigma_{V_{k-i}})\\ \;\;\;\;\;\;=\frac{1}{N}\sum_{i=1}^{N}A_{k-i}\Sigma_{{\hat{X}}_{k-i}}A_{k-i}^{T}+\frac{1}{N}\sum_{i=1}^{N}(V_{k-i}-x_{1}){\left(V_{k-i}-x_{1}\right)}^{T}\\ \;\;\;\;\;=\frac{1}{N}\sum_{i=1}^{N}A_{k-i}\Sigma_{{\hat{X}}_{k-i}}A_{k-i}^{T}+x_{4} \end{array}$$

As the expectation of the predicted state residual is zero, i.e.,

(29) $$E({V^{\prime }}_{{\bar{X}}_{k-i}})=0$$

then, by (13), we can obtain

(30) $$\begin{array}{l} {\hat{s}}_{k}=\frac{1}{N}\sum_{i=1}^{N}\left({\hat{X}}_{k-i}-{\bar{X}}_{k-i}\right)\\ \;\;\;\;=\frac{1}{N}\sum_{i=1}^{N}V_{{\bar{X}}_{k-i}}\\ \;\;\;\;=x_{2} \end{array}$$

By (12), we have

(31) $$\Sigma_{{\bar{X}}_{k-i}}=\Sigma_{V_{{\bar{X}}_{k-i}}}+\Sigma_{{\hat{X}}_{k-i}}$$

and

(32) $$\Sigma_{{\bar{X}}_{k-i}}=\Phi_{k,k-i-1}\Sigma_{{\hat{X}}_{k-i-1}}\Phi_{k,k-i-1}^{T}+\Sigma_{W_{k-i}}$$

Thus, we have

(33) $$\begin{array}{l} {\hat{\Sigma }}_{W_{k}}=\frac{1}{N}\sum_{i=1}^{N}\Sigma_{W_{k-i}}\\ \;\;\;\;\;\;\;\;=\frac{1}{N}\sum_{i=1}^{N}(\Sigma_{V_{{\bar{X}}_{k-i}}}+\Sigma_{{\hat{X}}_{k-i}}-\Phi_{k,k-i-1}\Sigma_{{\hat{X}}_{k-i-1}}\Phi_{k,k-i-1}^{T})\\ \;\;\;\;\;\;\;\;={\hat{\Sigma }}_{V_{{\bar{X}}_{k}}}+\frac{1}{N}\sum_{i=1}^{N}\Sigma_{{\hat{X}}_{k-i}}-\frac{1}{N}\sum_{i=1}^{N}\Phi_{k,k-i-1}\Sigma_{{\hat{X}}_{k-i-1}}\Phi_{k,k-i-1}^{T} \end{array}$$

Thus, the output $y_{2}$ of the fuzzy logic may be written as

(34) $$\begin{array}{l} y_{2}=\frac{1}{N}\sum_{i=1}^{N}\Sigma_{{\bar{X}}_{k-i}}\\ \;\;\;\;\;=\frac{1}{N}(\sum_{i=1}^{N}V_{{\bar{X}}_{k-i}}V_{{\bar{X}}_{k-i}}^{T}+\sum_{i=1}^{N}\Sigma_{{\hat{X}}_{k-i}})\\ \;\;\;\;\;={\hat{\Sigma }}_{{\hat{X}}_{k}}+x_{3} \end{array}$$

**Step 6**. Substituting (28) and (34) into the Kalman filtering framework, the system state estimation can be obtained.

**Figure 2:**
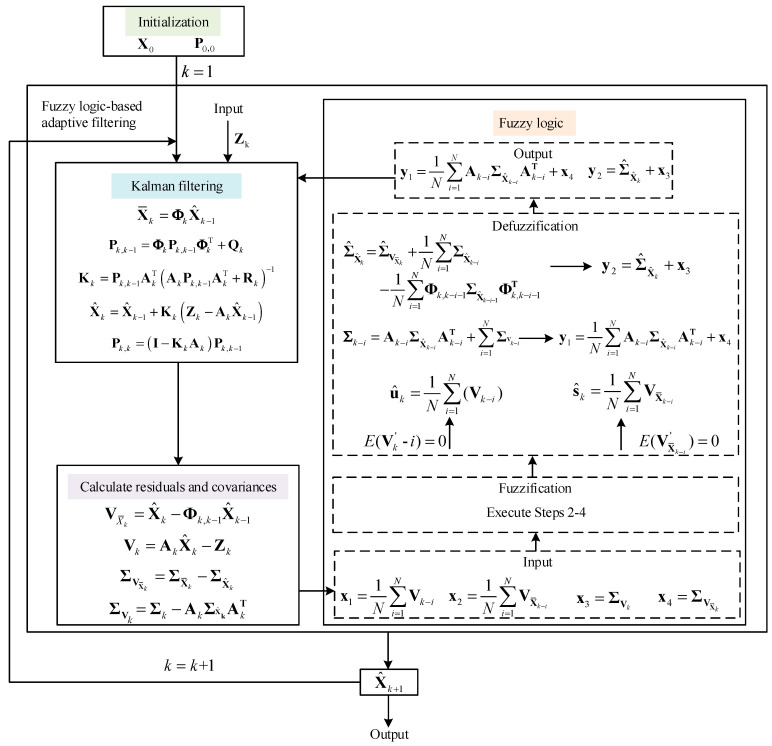
The framework of the fuzzy logic-based adaptive Kalman filtering algorithm.

Figure 2 illustrates the framework of the proposed fuzzy logic-based Kalman filter. It can be seen that the fuzzy logic module takes the measurement vector’s and predicted state’s residuals together with their covariances as input and adaptively outputs the measurement vector’s and predicted state’s covariances (y1 and y2) through the fuzzy logic rules. These outputs are further fed back to the Kalman filtering process to adaptively adjust the state error covariance $P_{k,k-1}$ and the Kalman gain $K_{k}$ to obtain the estimated state ${\hat{X}}_{k+1}$. After recursive iterations, the estimated state is eventually obtained and output as the alignment result.

## 5. Performance Evaluation and Discussion

Simulations and experiments on SINS transfer alignment were conducted for the performance evaluation of the proposed fuzzy logic-based adaptive Kalman filter (FLAKF). Comparison analysis with the Kalman filter (KF) and RAKF [40] were also conducted to demonstrate the enhanced performance of the proposed FLAKF.

### 5.1. Simulation and Analysis

**Figure 3:**
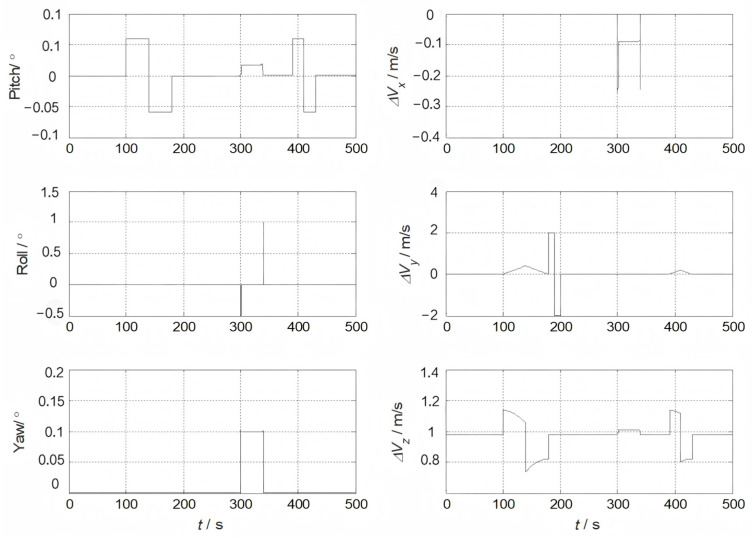
The aircraft’s dynamic motion.

The performance evaluation of the proposed method was evaluated by simulating the navigation of an aircraft with an airborne SINS for transfer alignment. The dynamic trajectory of the aircraft is shown in Figure 3. The aircraft involves various maneuvers such as climbing, acceleration, deceleration, level flight, and turning, provided in Table 1.

The SINS was located at latitude 34.2° N, longitude 108.9° E, and height 2000 m. The initial state was set to zero, the accelerometer bias was $1\times 10^{-4}\;g$, the gyro constant drift was $0.01^{\circ }/h$, the measurement noise of velocity was 0.2 m/s with the covariance matrix of ${\left(0.2\;m/s\right)}^{2}I_{2\times 2}$, the angular measurement error was 1 arcmin with the covariance matrix of ${\left(1\;arc\min\right)}^{2}I_{3\times 3}$, the sampling cycle was 0.1 s, and the simulation time was 500 s. The reference misalignment angles in the East, North, and Up directions were set as 10 arcmin, −10 arcmin, and 50 arcmin, respectively.

To assess the ability of the proposed method in suppressing the influence of system model error on state estimation, a constant system model error of 0.03 was added to both the predicted state and measurement within the time period from 8 s to 32 s. For comparison purposes, simulation trials were conducted under the same conditions by KF, RAKF, and FLAKF.

**Figure 4:**
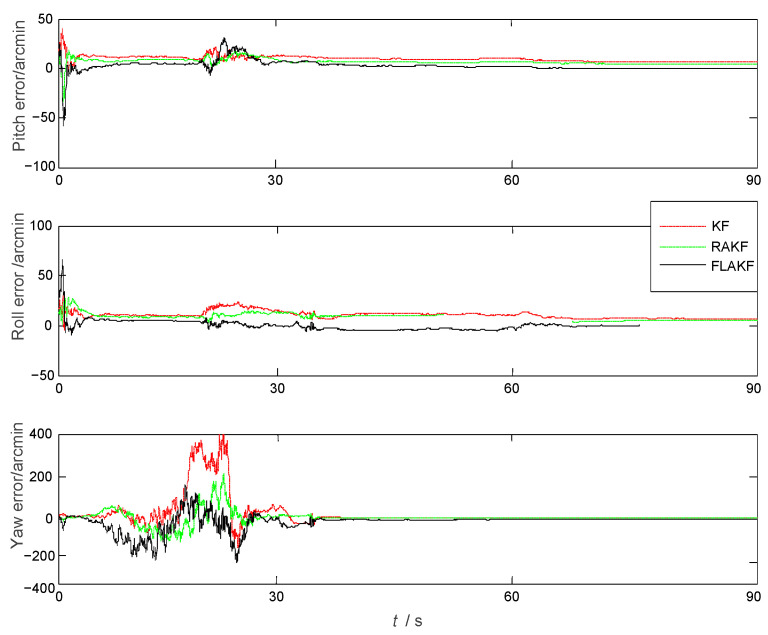
The attitude errors obtained by KF, RAKF, and FLAKF.

**Figure 5:**
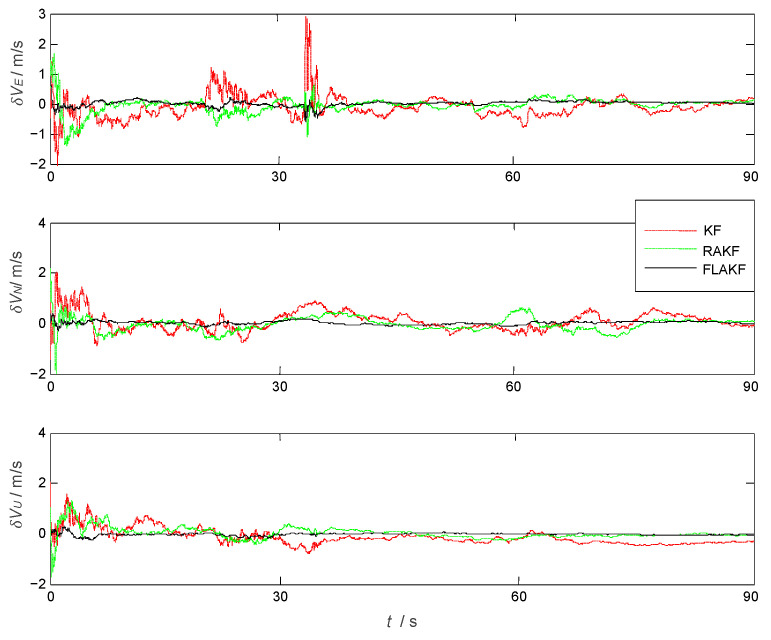
The velocity errors obtained by KF, RAKF, and FLAKF.

**Figure 6:**
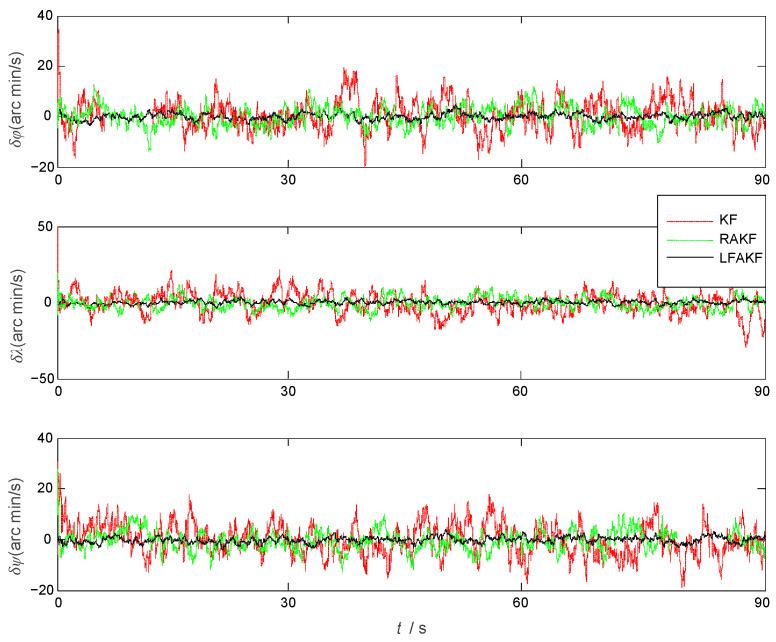
The position errors obtained by KF, RAKF, and FLAKF.

The position velocity and attitude errors of the aircraft by KF, RAKF, and FLAKF are shown in Figure 4, Figure 5 and Figure 6, respectively. As we can see from Figure 4, in the initial stage, due to the influence of atmospheric current, all three approaches had obvious errors in pitch and roll. KF had the largest error in yaw, which was more than 300 arcmin. After stabilization, the errors of all the methods were significantly reduced. As shown in Figure 6, the velocity error also had a similar trend to the attitude error. KF had the largest velocity error, which was within ±0.6 m/s, while FLAKF had the smallest velocity error, which was within ±0.1 m/s. Despite the improved accuracy compared to KF, RAKF still involved an obvious velocity error, which was within ±0.4 m/s. Similar to the attitude and velocity errors, as shown in Figure 6, the position error of FLAKF was also smaller than those of KF and RAKF.

As shown in Figure 4, the system model error mainly impacted the yaw angle error within (10 s, 32 s), while its impacts on the velocity errors in the North and Up directions were relatively small. FLAKF suppressed the system model error’s impact, leading to the smaller yaw angle error compared to KF and RAKF. A similar trend can also be observed in Figure 5. The system model error had a large impact on the velocity error in the East direction within (20 s, 35 s), while there was a relatively small impact on the velocity errors in the North and Up directions. FLAKF suppressed the system model error’s impact, leading to the smaller yaw angle error compared to KF and RAKF. Table 2 shows the statistical errors of the three methods. It can be seen that the mean errors in pitch, roll, and yaw estimated by FLAKF were 1.5398 arcmin, 1.8346 arcmin, and 15.2796 arcmin and the standard deviations were 2.5523 arcmin, 2.2118 arcmin, and 34.1316 arcmin, respectively. The above results demonstrate that FLAK can effectively suppress the interference of system model error in comparison with KF and RAKF.

### 5.2. Experimental Analysis

For further evaluation performance of the proposed FLAKF, experiments were also conducted for the transfer alignment of a missile launched with an ASN 206 reconnaissance UAV (Unmanned Aerial Vehicle). This UAV was equipped with a SINS (master) and the missile was equipped with a small airborne SINS (slave). Table 3 presents the parameters of the master and slave SINS.

**Figure 7:**
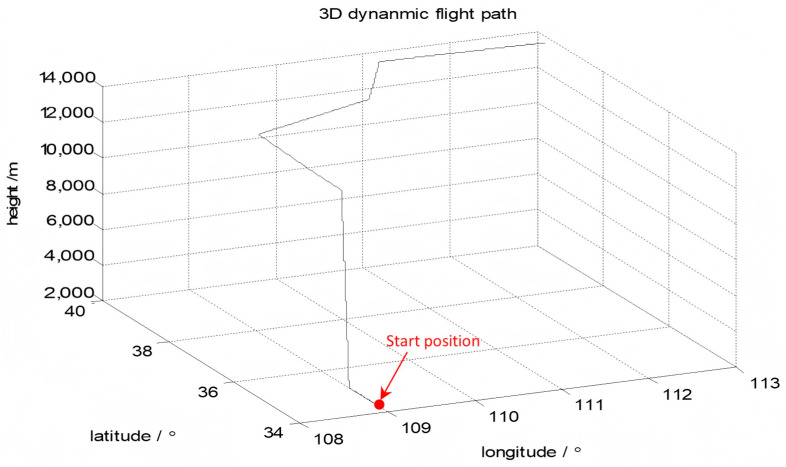
UAV flight trajectory.

The UAV lifted off after the five-minute initialization. The flight time was 30 min. The flight trajectory of the UAV is shown in Figure 7. The initial position of the UAN was North latitude 35.237°, East longitude 109.013°, height 2.8 km. The initial heading angle was φ=0°, the initial pitch angle was $\beta =0^{{}^{\circ}}$, and the initial roll angle was γ=0°; the initial misalignment angles were $\Phi_{E}=1{}^{\circ},\;\Phi_{N}=1{}^{\circ}\;\mathrm{and}\;\Phi_{U}=1{}^{\circ}$. The constant bias of the accelerometer was $1\times 10^{-4}\;g$ and the Gyro constant drift was 0.1(°)/*h*. The covariance matrix of the initial state was

(35) $$P_{0}=diag\left(\begin{array}{cccccc} P_{v,i} & P_{\phi ,j} & P_{\delta \varphi } & P_{\delta ,\lambda } & P_{\nabla ,i} & P_{\varepsilon ,j} \end{array}\right)$$

where $P_{v,i}={\left(0.2\;m/s\right)}^{2}$, $P_{\phi ,j}={\left(1\;arc\min\right)}^{2}$, $P_{\delta \varphi }=P_{\delta \lambda }=0.1^{{}^{\circ}}$, and $P_{\nabla ,i}={\left(2\times 10^{-4}\;g\right)}^{2}$.

The UAV flapped its wings and flew at an angle of $-15^{\circ }\sim 15^{\circ }$ for 1500 s, maintaining a horizontal uniform speed of 300 m/s throughout the entire maneuvering process. During the time period 0~15 s, the carrier aircraft tilted to the right fly at roll angular velocity γ=15°/s. During the time period 16~30 s, the carrier aircraft tilted to the left fly at roll angular velocity γ=15°/s. Within the time period 31~45 s, the carrier aircraft tilted to the right fly at the roll angular velocity γ=15°/s. During the time period 46~60 s, the carrier aircraft tilted to the left fly at roll angular velocity γ=15°/s. Within the time period (61 s, 90 s), the carrier aircraft resumed level flight. The experimental data within 90 s was used for estimating the misalignment angles. The filtering period was 1 s. It should be noted that the system model error, which was the deviation between the theoretical model and the actual system, was unknown.

**Figure 8:**
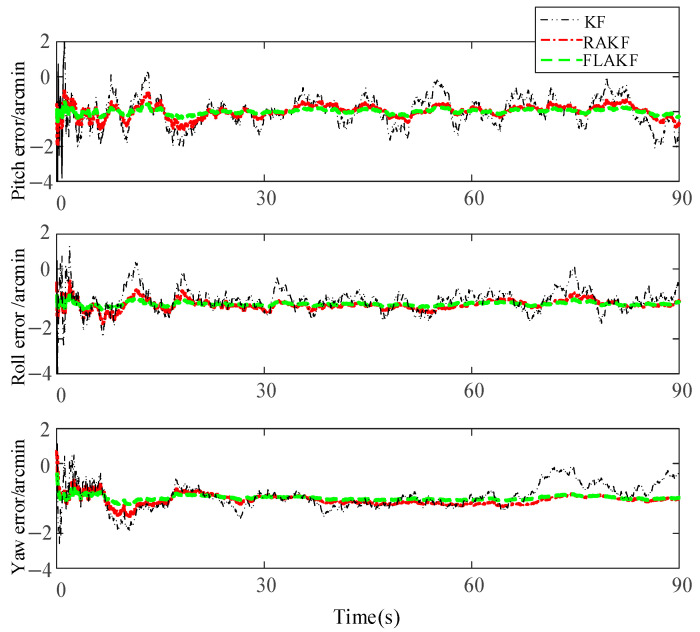
Misalignment angle errors of KF, RAKF, and FLAKF for the experimental case.

Trials on the transfer alignment were conducted by KF, RAKF, and FLAKF. Figure 8 shows the SINS misalignment angle estimation errors calculated by KF, RAKF, and FLAKF. From Figure 8, it can be seen that these three methods had large pitch, roll, and yaw angle errors in the initial stage, which gradually converged after approximately 25 s. The average errors of the misalignment angles of KF and RAKF were −4.058 arcmin and −3.905 arcmin and their standard deviations were 3.623 arcmin and 1.683 arcmin, respectively. The estimation error of the misalignment angle by FLAKF was within (−1.8 arcmin, +1.8 arcmin) and (−1.3 arcmin, +1.3 arcmin), respectively.

Compared with KF and RAKF, the error estimation curve of FLAKF for the SINS misalignment angle did not involve any obvious fluctuations. The FLAKF estimation errors of the pitch, roll, and yaw misalignment angles converged within 75 s. The fluctuations in the FLAKF estimation curve were within (−3.0 arcmin, 0.3 arcmin), which were significantly smaller than those in the KF and RAKF estimation curves. Table 4 shows the misalignment angles errors estimated by KF, RAKF, and FLAKF.

## 6. Conclusions

This paper presented an adaptive fuzzy logic-based Kalman filter to inhibit the influence of system model error on state estimation for transfer alignment. This method established an airborne SINS transfer alignment error model and introduced the fuzzy theory in the Kalman filtering process to estimate the covariance matrices of system measurement and predicted state. Through simulations and experimentation together with comparative analyses, it was proven that the proposed method can effectively suppress the influence of system model error on transfer alignment, leading to at least 18.83% higher accuracy for transfer alignment compared to benchmark methods.

Further research work will focus on the improvement of the proposed method. In future, the proposed method will be combined with advanced statistical computation methods such as the random weighting estimation [46] and set membership [47] to handle the random signals and uncertainties involved in the fuzzy theory for further improvement of transfer alignment accuracy.

## Figures and Tables

**Table 1:** The aircraft maneuvers.

Stage	State	Time (s)	Pitch (Deg/s)	Roll (Deg/s)	Yaw (Deg/s)	Acceleration (m/s^2^)
1	Level flight	100	0	0	0	0, 0, 0
2	Climbing	40	0.6	0	0	0, 0, 1.575
3	Level off	40	−0.6	0	0	0, 0, −1.575
4	Acceleration	10	0	0	0	0, 20, 0
5	Deceleration	10	0	0	0	0, −20, 0
6	Level flight	100	0	0	0	0, 0, 0
7	Turn left	2	0	−5	1	−2.625, 0, 0
8	Turning phase	37	0	0	1	−2.625, 0, 0
9	Level off	1	0	10	1	−2.625, 0, 0
10	Level flight	50	0	0	0	0, 0, 0
11	Climbing	20	0.6	0	0	0, 0, 1.575
12	Level off	20	−0.6	0	0	0, 0, −1.575
13	Level flight	70	0	0	0	0, 0, 0

**Table 2:** Statistical results of the attitude errors obtained by KF, RAKF, and FLAKF.

Method	Error Type	Pitch	Roll	Yaw
KF	Mean error	2.4399	2.7870	20.9531
	Standard deviation	4.5586	3.7869	39.9726
RAKF	Mean error	2.3131	2.4166	18.3715
	Standard deviation	3.2076	3.3075	39.7953
FLAKF	Mean error	1.5398	1.8346	15.2796
	Standard deviation	2.5523	2.2118	34.1316

**Table 3:** The parameters of the master and slave SINSs.

Sensor	Error Sources	Values (1σ)
Master SINS	Gyro constant drift	0.01(°)/*h*
	Initial deviation of the accelerometer	$10^{-5}\;g$
Slave SINS	Gyro constant drift	0.1(°)/*h*
	Initial deviation of the accelerometer	$10^{-3}\;g$

**Table 4:** Estimation errors of the misalignment angle (arcmin) by KF, RAKF, and FLAKF for the experimental case.

Filtering Method	Average Error	Standard Error	Misalignment
KF	−4.058	3.623	(−1.8, +1.8)
RAKF	−3.905	1.683	(−1.3, +1.3)
FLAKF	0.295	0.251	(−0.3, +0.3)

## Data Availability

The data are available from the lead author upon reasonable request.
